# Disinfecting Means and Methods

**Published:** 1890-11

**Authors:** 


### Disinfecting Means and Methods.

The following abstract is from the *Centralblatt fur die Med. Wissensch.* (No. 30, 1890): In an earlier paper, Herr Geppert had stated that "sublimate" has not the disinfecting action usually ascribed to it (*Centralb. f. d. Med. W.*, No. 9, 1890). In pursuance of his investigations, Geppert shows that its strong antiseptic action in earlier experiments lay in the substratum (holding the spores) which served as indicator, viz., threads or cotton-wool fibres. A kind of chemical combination occurs, so that the most careful washing with water, as generally practiced, can not remove the perchloride of mercury; hence the growth of the spores is prevented on nutrient media, and a true disinfection is simulated. When, instead of threads, steel needles or covering glasses were used, and touched with anthrax spores, the latter developed into a "culture," even after twenty minutes' immersion in a one per thousand sublimate solution, and inoculated animals died of splenic fever. In fact, in the use of sublimate, the impression falls on the object and not on the infection-carrier. The more such an object, loaded with sublimate, is in a condition to retain it, the less can it disinfect.

Also the concentrated (7 per cent.) watery solution of carbolic acid was examined similarly, and after thirty-eight days' stay in it, anthrax bacilli were still capable of growth and of infecting animals.

Boiling water was next examined. After one minute in this, spores were still able to develop and infect; after two minutes' boiling, spores could no longer infect animals, but could still develop in agar; it required five minutes' boiling to prevent subsequent growth. The result, repeatedly verified by the author, that growth upon nutrient media and the capability of infecting animals are not lost *pari passu*, induced him to take as his criterion of perfect infection the loss of action on animals. In the case of boiling water, the spores could grow after they were found to be harmless on animals, but in the case of sublimate the opposite occurred. Pieces of skin into which anthrax spores were rubbed, and which were dipped for five minutes in sublimate solution, showed no growth of the spores on agar, but inoculated rabbits, and mice died of splenic fever.

The strongest antiseptic was found to be the oldest we know of, viz., chlorine. A watery solution holding 0.2 per cent. of chlorine destroyed, in fifteen seconds, the infectiveness and power of growth of anthrax bacilli; at the moment of contact it so attenuates the spores that they are no longer able to kill rabbits, which are evidently not immune against splenic fever. On the other hand, the power of chlorine to prevent development is only slight; it requires a strength of one in 700, at least, to stop the development of anthrax spores on agar. In the nascent condition, chlorine is still stronger than aq. chlori. If to a weak mixture of chloride of lime and water, containing anthrax spores, HCl (three per cent.) was added to the latter, and then immediately weak $NH_3$, inoculated animals did not die, but if water was used *vice* HCl they died of splenic fever. Since in the former method of using chlorine the suffocating smell of chlorine is absent, the author selected this way of using chlorine as the best in medical practice. Moreover, by this method the previous use of soap and water is unnecessary, because the mixture is eminently cleansing and removes dirty layers of organic material. To cleanse the hands then, a paste is used of 100 parts of powdered chloride of lime with 45 of water; this is like an ointment. It is rubbed well over the hands, which are then dipped in a two or four per cent. solution of HCl; finally, the nails are gently brushed. If certainty of disinfection is desired, the hands may be previously dipped in gentian-violet solution; if the chlorine acts sufficiently, the color will be entirely discharged. Instead of the paste the hands may be dipped alternately in chloride of lime solution and HCl three per cent.

The author thinks it very important that in experiments where an infection-carrier is removed from a liquid, we should be able to cleanse it effectually from that liquid. This is not the case with threads; hence the source of error.—*London Med. Recorder.*

---

BORAX is highly recommended (in doses of from thirty to sixty grains) in cases of functional epilepsy.

